# Brain MR‐only workflow in clinical practice: A comparison among generators for quality assurance and patient positioning

**DOI:** 10.1002/acm2.14583

**Published:** 2024-11-25

**Authors:** Mathilde Levardon, Damien Autret, Thomas Le Dorze, Camille Guillerminet, Stéphane Dufreneix

**Affiliations:** ^1^ Department of Nuclear Medicine Centre Hospitalier Universitaire d'Angers Angers France; ^2^ Department of Medical Physics Institut de Cancérologie de l'Ouest Angers France

**Keywords:** artificial intelligence, brain, quality assurance, synthetic CT

## Abstract

**Background and purpose:**

Routine quality control procedures are still required for sCT based on artificial intelligence (AI) to verify the performance of the generators. The aim of this study was to evaluate three generators based on AI or bulk density (BD) assignment for the patient‐specific quality assurance (PSQA) of another AI‐based generator in clinical routine. A patient positioning study based on 2D/2D kV‐image comparing the performances of four sCT generators was also performed.

**Materials and methods:**

On the four generators available commercially at our institution, one was chosen as the clinical one, and the three others were used for PSQA. Several dose metrics were calculated like the mean error, dose‐volume histogram metrics, and 1%/1 mm gamma analysis. A comparison against CT was considered as a reference. Translations and rotations found during patient positioning based on sCT were compared to those based on CT.

**Results:**

Some of the metrics calculated against CT revealed patients outside the tolerances chosen (1% for point metrics; 90% for gamma pass rate). None of the generators was able to identify these outliers for all metrics studied. Performing a PSQA with other sCT generators introduced several false positives and false negatives. None of the generators was able to clearly identify, for all metrics studied, a true sCT failure caused by a metal implant. The smallest positioning deviations were found for the BD assignment sCT, the largest for the only AI generator not based on a T1 Dixon MR sequence.

**Conclusions:**

PSQA of a sCT generator with another sCT generator should be performed with great care. Patient positioning is an important aspect to consider when evaluating a sCT generator. The results of this study should help medical physicists willing to set up a MR‐only workflow for the brain based on a 2D/2D kV‐image patient positioning.

## 1 INTRODUCTION

The interest in MR‐only workflows has grown with the introduction of synthetic CT (sCT) generators based on artificial intelligence (AI). These images are generated from MR images and allow to perform dose calculation, without a CT exam. sCT are now widely used^1^, ^2^ for various locations and several generators were validated, especially for the brain.^3^, ^4^, ^5^, ^6^ Generators are commonly validated by comparing sCT to CT^7^ in terms of Hounsfield Unit (HU) and dosimetry. In an MR‐only workflow, however, no CT is available to perform a patient‐specific quality assurance (PSQA) and the sCT can not be individually validated for each patient. Routine quality control procedures are still required for AI‐based sCT to verify the performance of the generator regularly.^8^, ^9^

The most common approach to validate a sCT is a visual inspection to ensure the absence of artifacts.^10^, ^11^ It was also suggested to evaluate a sCT generated from MR images against another sCT generated from CBCT images used for patient repositioning. This was performed for prostate^12^, ^13^ and head & neck.^14^ For the brain, however, this approach can be limited if no CBCT is performed, for example when patient positioning is performed with the Exactrac (Brainlab) system based on two kV images. An alternative approach is then to evaluate a sCT generator based on MR images against another one, also based on MR images. This was performed in two studies for the pelvis^15^ and abdomen^16^ but not for the brain. The first aim of this study was to extend this approach for PSQA in the brain using commercially available sCT generators based on MR images. Three generators based on AI or bulk density (BD) assignment were evaluated for the PSQA of another AI generator dedicated to clinical routine.

In addition to setting up a robust PSQA program, another critical aspect when implementing a MR‐only workflow is patient positioning. For the brain, this aspect is often either overlooked^1^, ^4^, ^17^ or studied based on cone beam CT (CBCT).^18^, ^19^ Only one study^5^ evaluated patient positioning for a single commercial sCT generator for the Exactrac positioning system (Brainlab). The second aim of this study was thus to compare, on the same patient cohort, the performances of four sCT generators commercially available for the brain in terms of patient positioning based on 2D/2D kV‐images.

## 2 MATERIALS AND METHODS

### 2.1 Patient cohort

This study was based on data collected during a clinical study approved by the Angers Ethics Committee aiming at validating various sCT generators.^4^, ^20^ Forty two patients were enrolled with various diseases (glioma: 81%, meningioma: 12%, astrocytoma: 5%, and carcinopharyngioma: 2%). Both genders were equally represented and median age was 66 years (35–84y). Patients underwent MR exam on a 1,5 T Siemens Magnetom Aera XJ MRI scan and CT exam on a Siemens Confidence RT scan (120 kV, 2 mm slice thickness). Both exams were performed in radiotherapy treatment conditions including a thermoplastic three‐point mask. One of the patients (patient number 14) had a metal implant generating an artifact on the MR images and served as a true failure in the case of PSQA.

### 2.2 Generators

sCTs were generated for each patient by four commercially available softwares:

1. Syngovia (Siemens Healthineers), based on a T1 DIXON sequence and a densely connected UNet generator associated with a conditional Generative Adversarial Network. This sCT, denoted sCT_clin_ was arbitrarily chosen as the “clinical” one and consequently, the one to be evaluated against the others.
2. MRPlanner (Spectronic Medical AB), based on a T1 DIXON sequence and a deep‐learning‐based Transfer Function Estimation algorithm. sCT from this generator were denoted sCT_QA_AI_1_. The comparison between sCT_clin_ and sCT_QA_AI_1_ evaluated if using a different generator based on the same MR sequence was efficient for PSQA. This generator automatically rejected patients with artifacts on the MR image. Its associated patient cohort is thus smaller than that of the other generators.
3. ART‐Plan (Therapanacea), based on a T1 MPRAGE Gadolinium 3D sequence and an end‐to‐end ensembled self‐supervised Generative Adversarial Networks endowed with cycle consistency. sCT from this generator were denoted sCT_QA_AI_2_. The comparison between sCT_clin_ and sCT_QA_AI_2_ evaluated both the change of MR sequence and the change of AI generator in view of PSQA.
4. Syngovia (Siemens Healthineers), based on several sequences: T2 Space 3D, a T2 PETRA 3D (Bones), a T1 DIXON 3D, and a T2 FLASH Gradient Echo 2D (Vessels). Contrary to previous AI‐based generators, this one, denoted sCT_QA_BD_ was based on a BD method.

**FIGURE 1:**
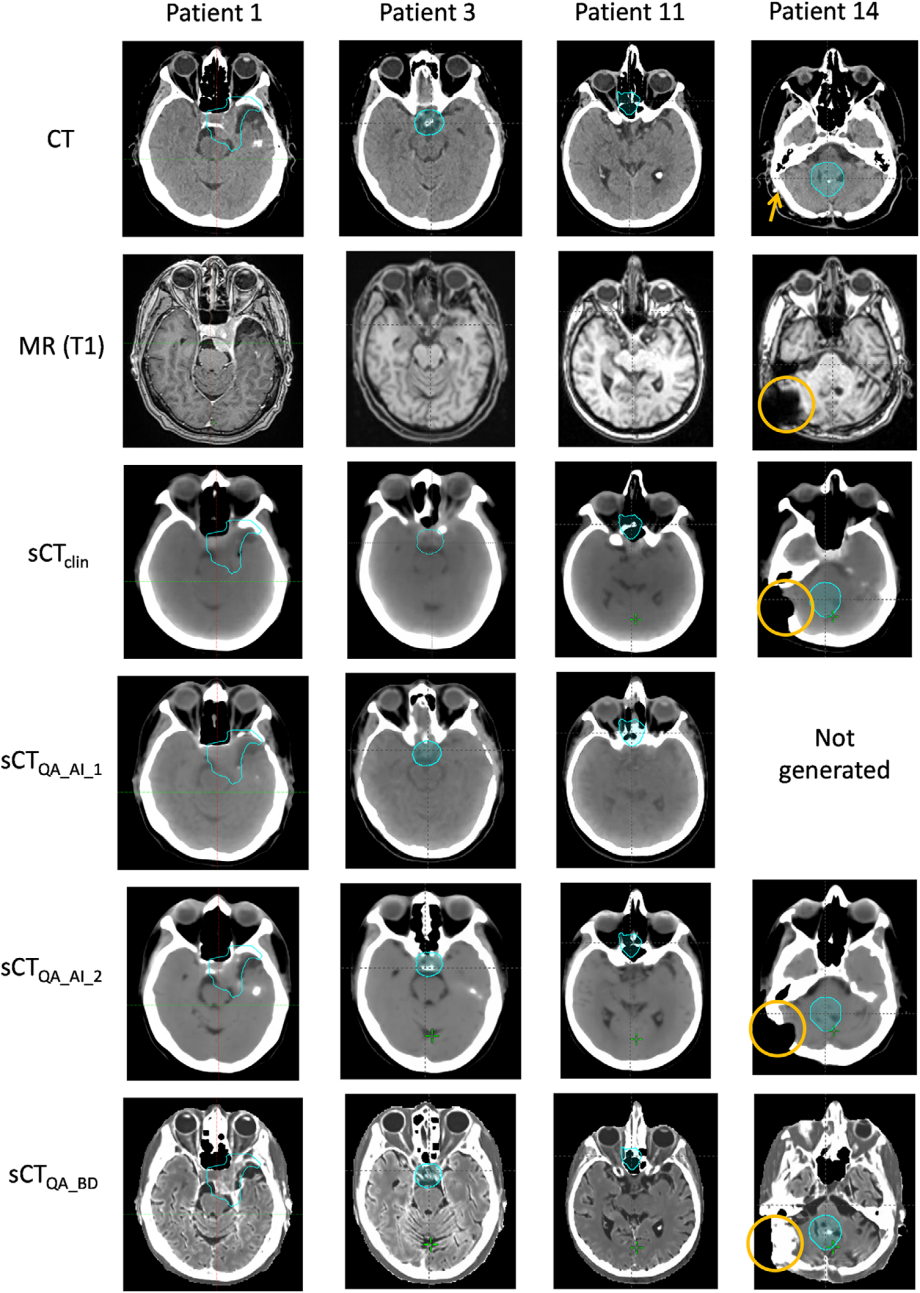
Example of axial images for the CT, MR, and sCTs studied (viewing window for CT and sCT: [− 20; 100] HU). PTV is shown in cyan. For patient 14, the metal implant and its artifact are highlighted in orange. PTV, planning target volume; sCT, synthetic CT.

Some examples of CT and sCT images for four patients are given in Figure 1. Patients 1, 3, and 11 illustrate the various predictions of sCT in the sinus region. Patient 3 additionally illustrates the reconstruction of a post‐operative skull (on the left side of the patient). Management of MR artifacts is illustrated with patient 14. All four sCT generators were evaluated against a CT in terms of image quality and dose calculation accuracy in a previous study.^4^

### 2.3 PSQA dose evaluation

Dose was optimized and calculated for the treatment plan on the CT with the AcurosXB algorithm (Varian Medical Systems) in dose to medium. Structures and plan were copied from the CT onto the four sCTs and dose was recalculated on the sCTs with the monitor units unchanged. Dose metrics were calculated for three structures: the planning target volume (PTV) delineated by the physician, the whole brain and the skull generated by thresholding the CT image ([−100; 100] HU for brain and ≥ 100 HU for skull). To compare sCT_clin_ to sCT and to CT, a rigid registration was performed.

A Python code (v3.11.5) was developed to calculate various metrics commonly used for sCT analysis.^21^, ^22^ The mean error inside each structure was calculated for various image sets (IS) considered as reference according to the formula:

$${\mathrm{ME}}^{\mathrm{structure}}\left(\mathrm{IS}\right)=\frac{1}{N}\;\sum_{i=1}^{N}D\left[{\mathrm{sCT}}_{\mathrm{clin},i}\right]-D\left[{\mathrm{IS}}_{i}\right]$$

where $D[{\mathrm{IS}}_{i}]$ is the dose value at voxel i for one of the following image sets: CT, sCT_QA_AI_1_, sCT_QA_AI_2_ or sCT_QA_BD_. This voxel‐to‐voxel metric is expressed in Gy and its ideal value is 0. A ME normalized to the prescribed dose and expressed in % was also calculated.

Dose‐volume histogram metrics were extracted using the library decompiler‐core v0.5.6^23^ and differences between ${\mathrm{sCT}}_{\mathrm{clin}}$ and (s)CT were computed according to the formula:

$$\Delta D_{x}^{\mathrm{structure}}\;\left(\mathrm{IS}\right)=\frac{D_{x}\left[{\mathrm{sCT}}_{\mathrm{clin},i}\right]-D_{x}\left[\mathrm{IS}\right]}{D_{\mathrm{pres}}}$$

where *D_x_* is either *D*_2%_, *D*_mean_, *D*_95%_ or *D*_98%,_ and *D*_pres_ is the prescribed dose.

Finally, a 3D local gamma analysis on the entire volume with a 20% threshold of the maximum dose with a 1% dose and 1 mm distance criteria was performed using the library PyMedPhys v0.39.3.^24^

Wilcoxon tests were performed to compare the distributions studied. Statistical significance was displayed on the figures with a discrete representation depending on the *p*‐value. Finally, the sensitivity of the various metrics was calculated according to the formula:

$$\mathrm{Sensitivity}=\frac{\mathrm{TP}}{\mathrm{TP}+\mathrm{FN}}$$

where TP is the number of True Positives and FN is the number of False Negative. Specificity was not calculated considering the small number of True Negative observed when validating the sCT_clin_ against CT.^4^

### 2.4 Patient positioning

**FIGURE 2:**
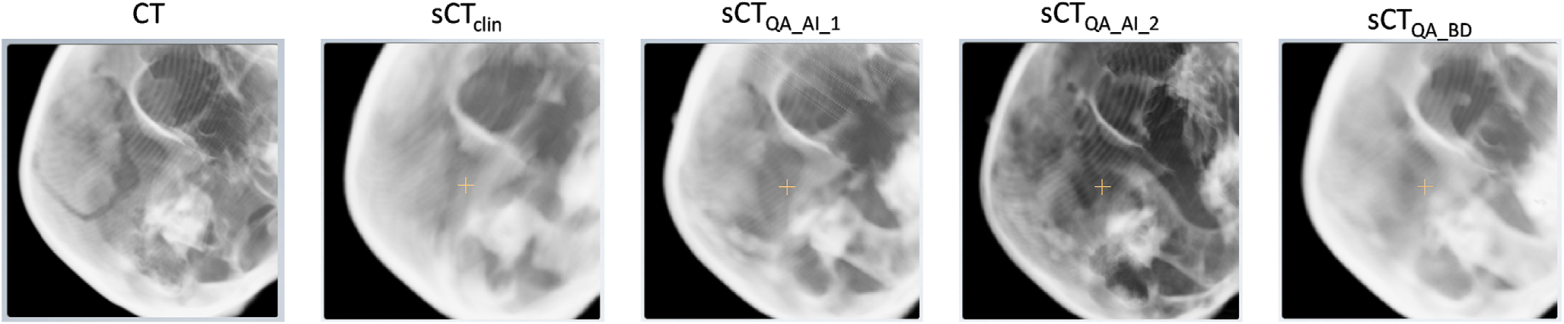
Example of DRR and sDRR. sDRR, synthetic digitally reconstructed radiographs.

Patients were treated on a Novalis Truebeam STx accelerator (Varian) equipped with the Exactrac system (Brainlab). A pair of two orthogonal kV images were compared to digitally reconstructed radiographs (DRR) generated from CT and resulting rotations and translations were applied. Synthetic DRR (sDRR) were retrospectively generated from sCT and the comparison between kV images and sDRR was performed for 3 fractions (one at the beginning, one in the middle and one at the end of treatment). Registrations were always performed in automatic mode based on bony structures. Examples of DRR and sDRR are provided in Figure 2. The difference of shifts/rotations between kV images and DRR on one hand and kV images and sDRR on the other hand was calculated. As mentioned by Masitho et al.,^5^ rotations between sCT and CT were subtracted to avoid a systematic error due to the CT/sCT registration and not related to patient positioning.

## 3 RESULTS

### 3.1 PSQA dose evaluation

**FIGURE 3:**
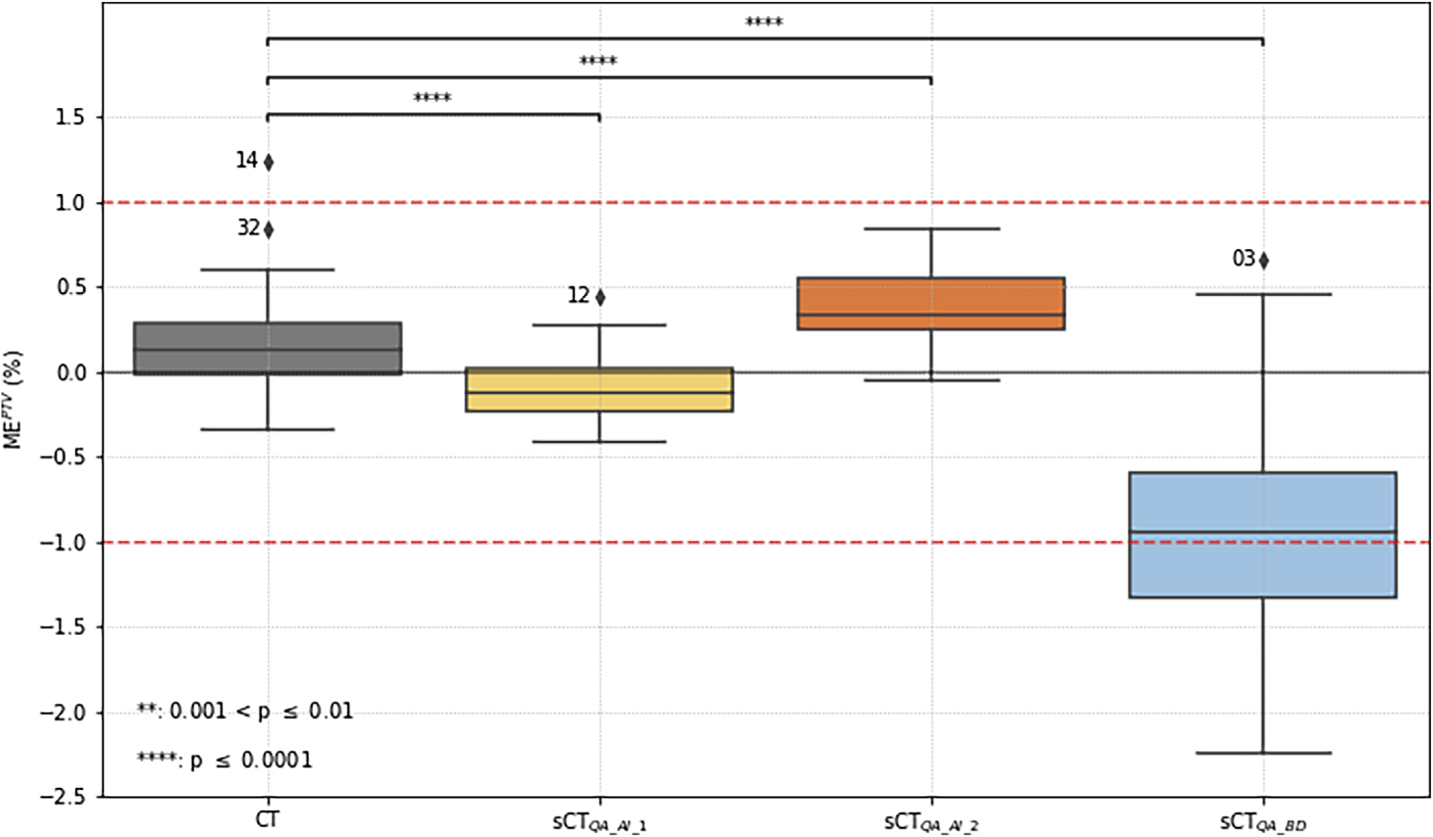
Boxplots of the mean error over the patient cohort for the PTV calculated for the sCTclin against other sCT and CT. Numbers next to outliers are the patient numbers. PTV, planning target volume; sCT, synthetic CT.

ME^PTV^ calculated against sCT_QA_AI_1_, sCT_QA_AI_2_, sCT_QA_BD_ and CT are plotted in Figure 3. ${\mathrm{ME}}^{\mathrm{PTV}}\left(\mathrm{CT}\right)$ was included in the [−1%; 1%] interval except for patient 14 with his metal implant. This outlier was not seen by the ${\mathrm{ME}}^{\mathrm{PTV}}\left({\mathrm{sCT}}_{\mathrm{QA}\_\mathrm{AI}\_1}\right)$ because the associated generator automatically rejected patients with artifacts on the MR image. It was however not seen by ${\mathrm{ME}}^{\mathrm{PTV}}\left({\mathrm{sCT}}_{\mathrm{QA}\_\mathrm{AI}\_2}\right)$ even if a sCT was generated. ${\mathrm{ME}}^{\mathrm{PTV}}\left({\mathrm{sCT}}_{\mathrm{QA}\_\mathrm{BD}}\right)$ revealed a −1% systematic difference. ME^Brain^ and ME^Skull^ are plotted in Figure S1. They were all included in the range [−0.4; 0.2] Gy corresponding to a deviation smaller than 0.5% of the prescribed dose. Considering a 1% tolerance, all PSQA would have been validated for sCT_QA_AI_1_ and sCT_QA_AI_2_ for the ME^PTV^ metric.

**FIGURE 4:**
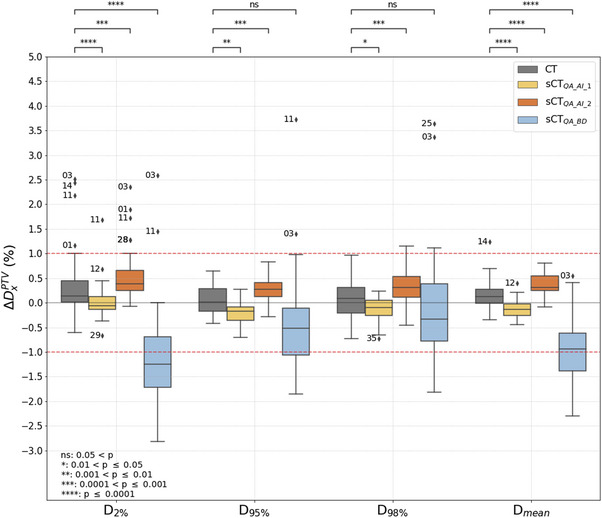
Boxplots of the DVH metric difference over the patient cohort for the PTV calculated for the sCTclin against other sCT and CT. Numbers next to outliers are the patient numbers. Numbers in blue indicate the number of outliers not shown. PTV, planning target volume; sCT, synthetic CT.

Dose‐volume histogram metrics are plotted in Figure 4. All $\Delta D_{x}^{\mathrm{PTV}}\;\left(\mathrm{CT}\right)$ were in the range of [−1%; 1%] except for patients 14, 11, and 3 regarding the *D*_2%_ and patient 14 regarding the *D*_mean_. Regarding the sCT_QA_AI_ generators, the most interesting metric was the *D*_2%_ because it revealed several outliers, some also observed for the CT (true negatives) but some not (false negatives). Considering a 1% tolerance, all PSQA would have been validated for the sCT_QA_AI_1_ generator and for all DVH metrics except patient 11 for the *D*_2%_. False positives were thus calculated for the sCT_QA_AI_1_ generator, like patient 3 for the *D*_2%_. Several false positives (patient 1 for the *D*_2%_ for example) and false negatives (patient 14 for the *D*_mean_ for example) were generated for the sCT_QA_AI_2_ generator. PSQA calculated with the sCT_QA_BD_ generator regularly stood above 1% and showed a systematic underestimation. $\Delta D_{x}^{\mathrm{Brain}}\;\left((s)\mathrm{CT}\right)$ and $\Delta D_{x}^{\mathrm{Skull}}\;\left((s)\mathrm{CT}\right)$ are plotted in Figure S2. Except for the D_2%_ where a difference of up to 4% could be observed, all metrics were smaller than 0.5%.

**FIGURE 5:**
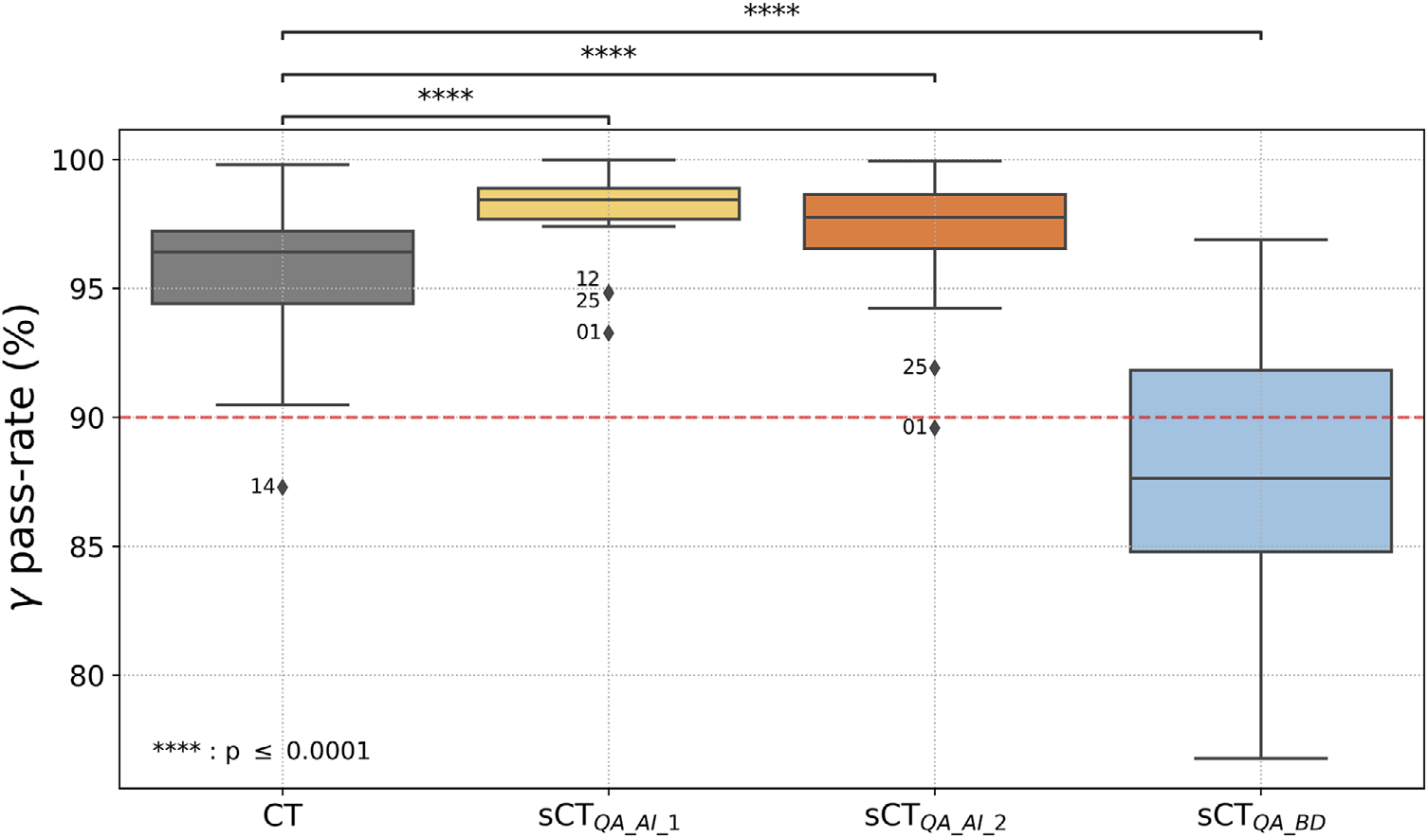
1%/1 mm gamma pass rates (local analysis on the entire volume with a 20% threshold).

1%–1 mm gamma pass rates are plotted in Figure 5. By considering an arbitrary 90% tolerance level, only one false positive (patient 1) was observed for sCT_QA_AI_2_ and none for sCT_QA_AI_1_. More than half of the PSQA generated with the sCT_BD_ generator were below this tolerance limit.

It can be seen on Figures 3, 4, 5 that no sCT distribution was statistically equivalent to the CT distribution, stressing the fact that sCTs can not be considered CT‐equivalent. Sensitivity of the previous metrics is given in Table 1. Both AI‐based generators gave sensitivity values close to 1. It should be recalled that sCT_QA_AI_1_ did not generate sCT for patients with artifacts on the MR image which can affect the results. Values for the BD assignment generator were much lower and showed that a simplistic sCT generator can not be used for PSQA in clinical routine, even if this was suggested in the literature.^9^

**TABLE 1:** Sensitivity of various metrics.

Metric	ME^PTV^	$D_{2\%}^{\mathrm{PTV}}$	$D_{95\%}^{\mathrm{PTV}}$	$D_{98\%}^{\mathrm{PTV}}$	$D_{\mathrm{mean}}^{\mathrm{PTV}}$	Gamma pass rate
Tolerance	±1%	±1%	±1%	±1%	±1%	> 90%
sCT_QA_AI_1_	1	1	1	1	1	1
sCT_QA_AI_2_	1	0.94	1	0.97	1	0.97
sCT_QA_BD_	0.56	0.32	0.66	0.68	0.54	0.32

Abbreviation: PVT, planning target volume.

### 3.2 Patient positioning

**FIGURE 6:**
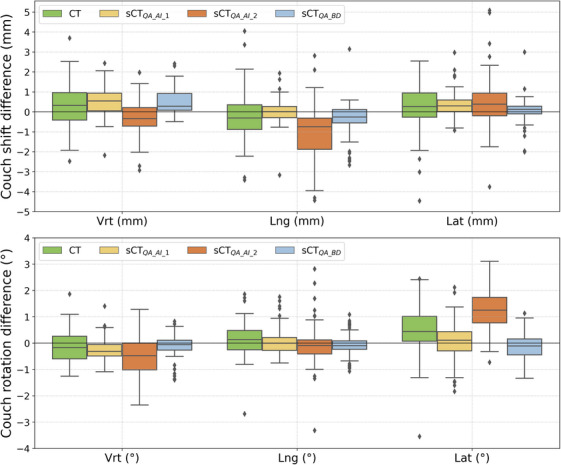
Couch shift differences (top) and couch rotation differences (bottom) in lateral, longitudinal, and vertical directions.

Boxplots of the shifts/rotations difference between kV images and DRR on one hand and kV images and sDRR on the other hand are displayed in Figure 6. Couch shifts up to 5 mm and couch rotations up to 4° were observed. The highest median shift difference (0.8 mm) was found in the longitudinal direction for the sCT_QA_AI_2_ and the highest median rotation difference (1.3 °) was found in the lateral direction for the same AI‐based generator. Median values for the sCT_clin_ were in agreement with those published by Masitho et al.^5^ Interestingly, sCT_QA_BD_ systematically showed the smallest median values as well as the smallest dispersion among the patients suggesting the BD assignment method is more adequate to generate 2D/2D sDRR.

## 4 DISCUSSION

This study evaluated if PSQA of a sCT generated in a MR‐only workflow could be performed with other sCT generators commercially available in clinical routine. sCT_QA_AI_1_ was found to be the most promising generator for PSQA: considering that it did not generate a sCT for patient 14 and his MR artifact, it could be considered as a good discriminator. However, some sCTs were also not generated because of dental artifacts located far from the PTV, limiting the use of this generator for PSQA. sCT_QA_AI_2_ was not able to discriminate patient 14 during any of the analyses conducted and its use for PSQA is thus mitigated, even if it can be argued that a visual inspection would have rejected patient 14 from a MR‐only workflow. The results of PSQA for both AI generators were significantly different from the comparison between sCT_clin_ and CT. The analysis could thus not distinguish the influence of the MR sequence (identical for sCT_clin_ and sCT_QA_AI_1_, different for sCT_QA_AI_2_) over the generator's architecture. sCT_QA_BD_ generated many positives in the case of PSQA but results were affected by the fact that it performed worse than sCT_clin_ when compared to a CT.^4^ Most of the patients were thus false positives. This analysis showed that a simplified sCT based on density assignment can not be used to validate an AI‐generated sCT.

Apart from patient 14 and the MR artifact, three patients (1, 3, and 11) showed a $\Delta D_{2\%}^{\mathrm{PTV}}\;\left(\mathrm{CT}\right)$ larger than 1% suggesting that the *D*_2%_ could be a robust metric to detect potential sCT failure. After investigations, it was found that the PTV of these patients was very close or inside the sinus region (Figure 1) characterized by its heterogeneity. As hypothesized by Vandewinckele et al.,^10^ differences between dose calculations based on various sCT revealed prediction difficulties.

Calculating sensitivity summarized the challenges of PSQA for sCT. It required the definition of a tolerance level for which no recommendation is yet available. For DVH endpoints, tolerance was set to 1% in this study but by Dal Bello et al.^16^ chose a 2% tolerance. The choice of the metric, its definition, and its associated tolerance limit should be clearly stated to define a True Negative. This issue is also met when comparing a sCT to a CT. In this study, no analysis or metric was robust enough to detect a true failure of the sCT (patient 14) and the best discriminator was a visual inspection of the sCT_clin_. Emin et al. recently described a similar procedure based on visual inspection of artifact occurrences for sCT quality assurance.^11^ Villegas et al. also stressed the importance of visual inspection to detect anatomic anomalies on the sCT.^9^ Such procedure remains to be detailed and will inevitably be only qualitative and subjective.

Patient positioning is another critical aspect when implementing a MR‐only workflow. Surprisingly, the best results were obtained for sCT_QA_BD_ although this generator performed worst against AI generators in terms of image quality and dosimetric evaluation.^4^ Worst results were found for the sCT_QA_AI_2_ generator which was the only one not to use a T1 DIXON MR sequence for sCT generation. This MR sequence is known to provide better bone contrast thus enhancing the reliability of bony structures in the sCT.^25^ sCT validation should include patient positioning based on the imaging protocol available because it can affect the choice of generator for clinical practice.

## 5 CONCLUSIONS

This study evaluated the feasibility of using various sCT generators to validate sCT available for clinical use in a MR‐only workflow for the brain. None of the generators based on AI or BD assignment were able to clearly identify, for all metrics studied, a real sCT failure caused by a metal implant. PSQA of a sCT in a MR‐only workflow remains challenging and results strongly depend on the metric studied. Patient positioning is also an important aspect to consider when implementing a MR‐only workflow and should be evaluated for the imaging system used in the clinical routine. The results of this study should help medical physicists willing to set up an MR‐only workflow for the brain associated with 2D/2D kV‐images patient positioning.

## AUTHOR CONTRIBUTIONS

Damien Autret, Camille Guillerminet, Mathilde Levardon, and Stéphane Dufreneix were responsible for the study design. Mathilde Levardon developed the Python code and Thomas Le Dorze adjusted it to the current study. Mathilde Levardon, Stéphane Dufreneix, and Damien Autret were responsible for data analysis and interpretation. Mathilde Levardon and Stéphane Dufreneix drafted the article.

## CONFLICT OF INTEREST STATEMENT

The authors declare no conflicts of interest.

## ETHICAL APPROVAL

Approval for the study protocol was obtained from the medical research ethics committee of CHU d'Angers and informed consent was obtained from all patients (ID RCB: 2020‐A01444‐35; Ref CPP: CPPIDF4 (Paris St Louis) 2020/72)

## Supporting information



Supporting Information.

Supporting Information.
