# Supplementary figures and images for: Brain MR‐only workflow in clinical practice: A comparison among generators for quality assurance and patient positioning
Source: J Appl Clin Med Phys. 2024 Nov 25;26(2):e14583. doi: 10.1002/acm2.14583 (PMC11799901; doi:10.1002/acm2.14583)

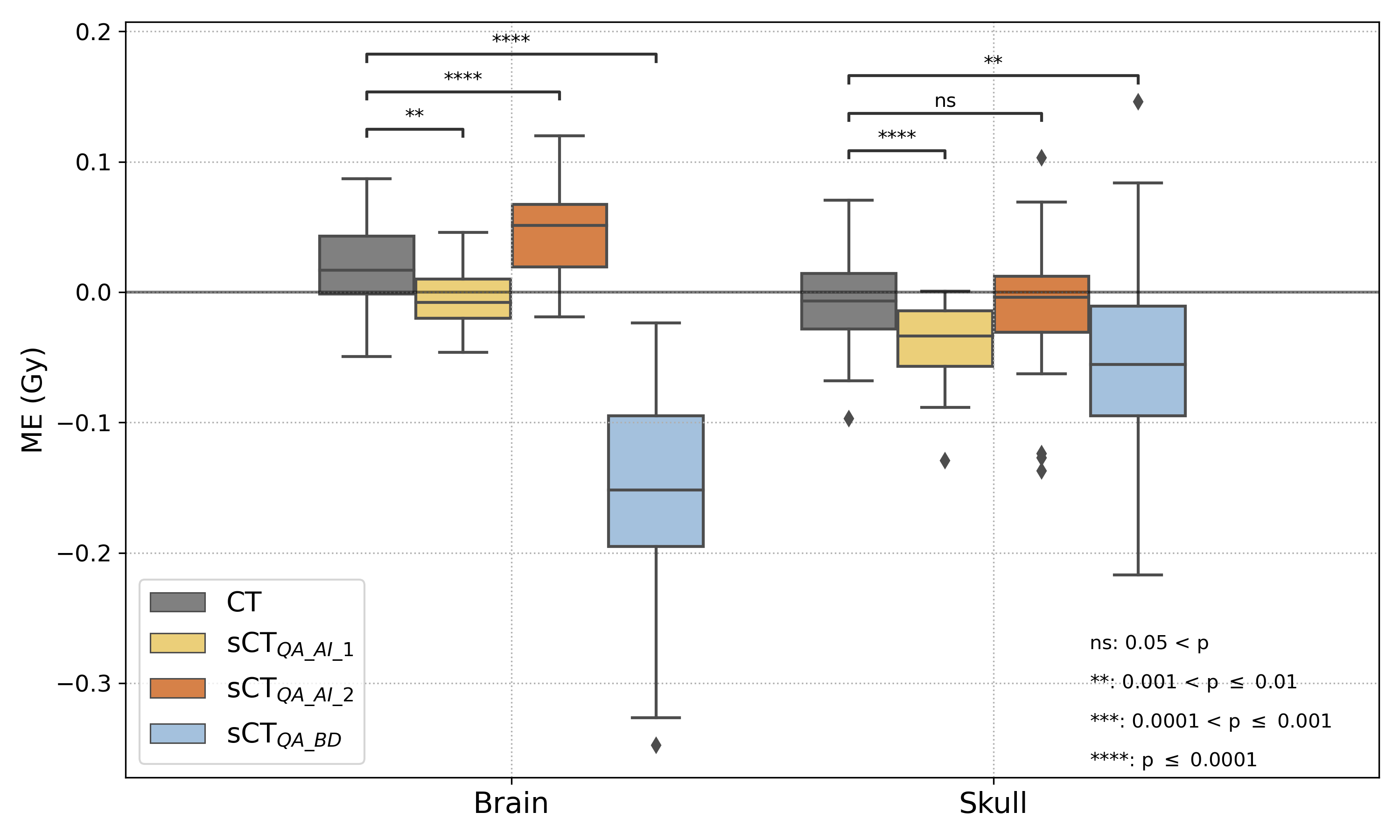

Supplement: Supplementary file 1 — Supporting Information. [file ACM2-26-e14583-s002.png]

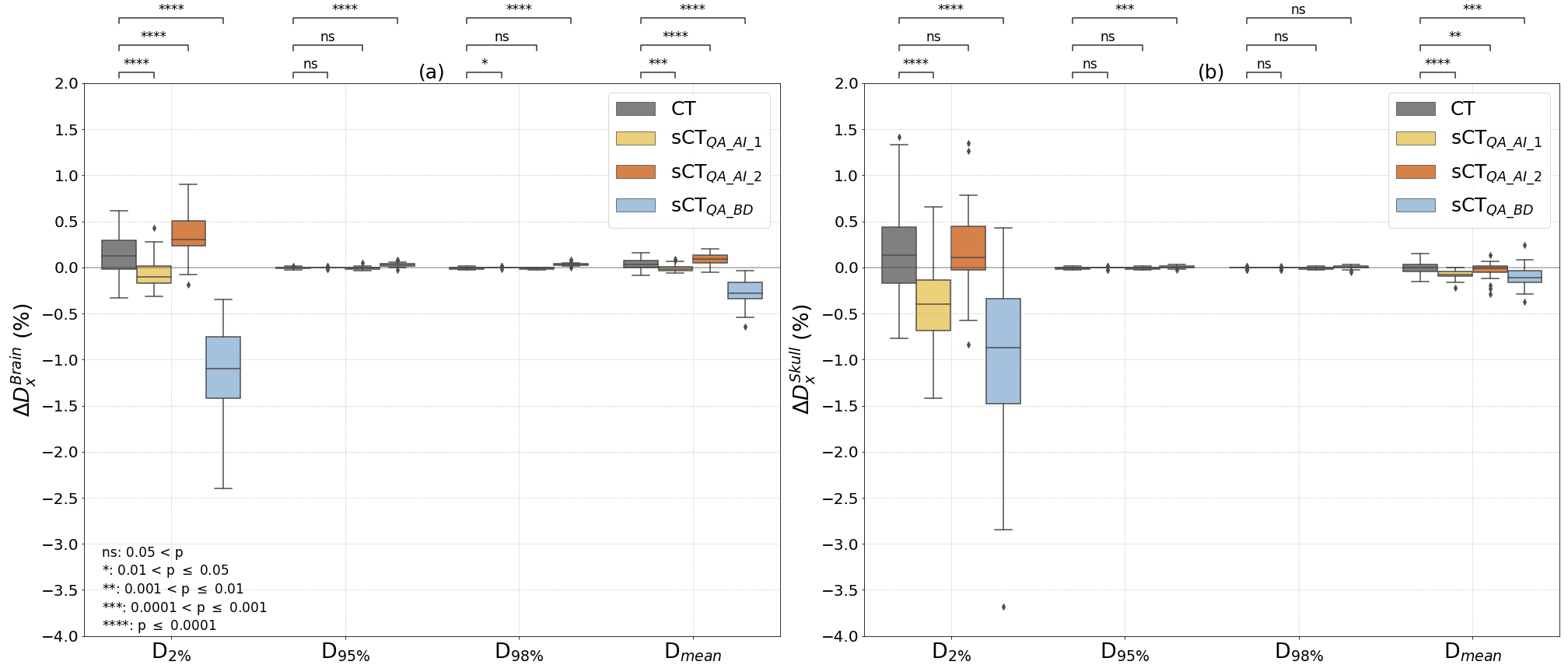

Supplement: Supplementary file 2 — Supporting Information. [file ACM2-26-e14583-s001.png]
